# *Chaenomeles sinensis* Koehne extract suppresses the development of atopic dermatitis-like lesions by regulating cytokine and filaggrin expression in NC/Nga mice

**DOI:** 10.7150/ijms.37854

**Published:** 2019-11-08

**Authors:** Kyung-Jae Cha, Chang-Seob Song, Ji-Sook Lee, Ayesha Kashif, Min Hwa Hong, Geunyeong Kim, In Sik Kim

**Affiliations:** 1 Department of Senior Healthcare, BK21 Plus Program, Graduate School, Eulji University, Daejeon 34824; 2 Happybio R&D center, Happybio, Cheongju, Chungcheongbuk-do 28101; 3 Department of Clinical Laboratory Science, Wonkwang Health Science University, Iksan, 54538; 4 Department of Biomedical Laboratory Science, School of Medicine, Eulji University, Daejeon 34824, Republic of Korea

**Keywords:** *Chaenomeles sinensis* Koehne, Anti-inflammatory effect, Atopic dermatitis, Filaggrin, JNK

## Abstract

*Chaenomeles sinensis* Koehne (CS) has been used in a traditional oriental medicine for treating throat diseases, anaphylaxis, viral infection, and inflammation. This study investigated the underlying mechanism of anti-allergic effect of CS. Leaves of CS plants were dried, powdered, and then underwent extraction with DMSO. Both ELISA and western blotting were performed to evaluate cytokine concentration and the expression and activation of filaggrin and JNK. Five-week‐old female NC/Nga mice were used as an AD-like mouse model by treating them with 2,4-dinitrochlorobenzene (DNCB). The secretion of TARC, MCP-1, and IL‐8 is increased by TNF-α and IFN-γ in HaCaT cells, and CS extract inhibited the increased production of TARC, MCP-1, and IL‐8. TNF-α and IFN-γ suppressed filaggrin expression by activating JNK. CS extract recovered the expression of filaggrin decreased by TNF-α and IFN-γ by blocking the activation of JNK. *In vivo* experiment, CS administration reduced thickening of the epidermis and infiltration of inflammatory cells into the dermis as compared to DNCB treatment. Moreover, the decrease of filaggrin expression due to DNCB treatment was recovered by CS administration. The serum IgE level was decreased by CS treatment. The levels of IL-4, IL-5, IL-13 and eotaxin in mouse splenocytes increased after treatment with concanavalin A, and the secretions of IL-4, IL-5, IL-13 and eotaxin were lower in the CS-treated group than in the DNCB group. These results may contribute to the development of a CS-based drug for the treatment of atopic dermatitis.

## Introduction

Atopic dermatitis (AD) is a chronic pruritic and inflammatory skin disease. The prevalence of AD has increased markedly in the world and is associated with hypersensitivity to environmental factors, allergens, immunological dysregulation, and genetic abnormality 1, 2. Approximately, 70% of patients suffering from AD show with extrinsic type of AD. They have increased serum IgE level, which contains antibody to a variety of food and allergens 3. AD is caused by an increase in T helper 2 lymphocytes and eosinophils, and by defect of skin barrier proteins including filaggrin, loricrin and involucrin 4-6. Filaggrin is an essential skin barrier protein in the epidermis with external and internal stimulation on the homeostasis of the skin 7, 8. Destruction of filaggrin is related to development and aggravation of AD, and such destruction results in inflammatory responses accompanied by erythema, itchiness, and scratching of the skin 9.

*Chaenomeles sinensis* Koehne (CS), a member of the Rosaceae family, is used as an herb in Korea and China for the treatment of throat diseases, anaphylaxis, viral infection, and neurodegenerative diseases 10-13. It has also been reported in anti- pruritic and anti-inflammatory activities and in the inhibition of oxidative damage due to free radical 14, 15. In this study, we investigated the inhibitory effect of CS on the expression of skin barrier proteins and cytokine secretion *in vitro* and *in vivo*.

## Materials and methods

### Preparation of CS extract

Whole CS plants (30g) were dried and incubated with DMSO for 24 h at room temperature. The complete CS extracts (voucher specimens No. 029-038) were used in the present study.

### Cell culture

Human keratinocytic HaCaT cells were cultured in Iscove's medium and Dulbecco's modified Eagle's medium supplemented with 10% fetal bovine serum and antibiotics. The cultured cells were maintained at sub-confluency in a 95% air, 5% CO_2_ humidified atmosphere at 37°C.

### MTT assay

Cell viability was assayed based on the conversion of 3-(4,5-dimethylthiazol-2-yl)-2,5-diphenyltetrazolium bromide (MTT) by using a cell proliferation kit (Roche Korea, Seoul, Korea). HaCaT cells in 100 μL culture medium were seeded into a 96‑well plate, and CS extract was added to the wells at concentrations ranging between 10 and 50 μg/mL. Following incubation for 48 h at 37°C, 10 μL MTT solution was added, followed by incubation for 4 h. Solubilization solution (100 μL) was added to the wells and following 24 h incubation, absorbance was measured at 550 nm by using an ELx808 enzyme‑linked immunosorbent assay (ELISA) reader (Bio‑Tek Instruments, Winooski, VT, USA).

### ELISA

After pre-stimulation with CS extract, HaCaT cells were treated with TNF‑α and IFN‑γ. Splenocytes were pre-treated with or without CS and then stimulated with 1 μg/mL concanavalin A (Merck, Kenilworth, NJ, USA) for 24 h and 48 h. Cell supernatants were collected and the concentrations of TARC, IL-6, IL‑8, MCP‑1, IL‑4, IL‑5, IL‑13, and eotaxin were measured in the supernatant by sandwich ELISA (BD Biosciences, San Jose, CA, USA and R&D Systems).

### Western blotting

The cells were lysed in CETi lysis buffer (TransLab) for 30 min, after which the lysate was centrifuged and the supernatant was collected. The total protein concentration of the lysate was evaluated using a BCA protein assay kit (Thermo Scientific) standardized to BSA, as per the manufacturer's instruction. Estimated cell lysates were then resolved by SDS-PAGE and transferred to nitrocellulose membranes. The blots were incubated with antibodies against filaggrin, phospho-JNK (Santa Cruz Biotechnology, Santa Cruz, CA, USA), involucrin, or loricrin (Proteintech, Rosemont, IL, USA), and subsequently incubated with HRP-conjugated secondary antibodies, including goat anti-rabbit or rabbit anti-mouse antibodies. Finally, the blots were developed using an enhanced chemiluminescence detection system (Thermo Scientific) and visualized by Chemidoc (Bio-Rad).

### Atopic dermatitis induction and CS administration in NC/Nga mice

Female* 5*-week‐old NC/Nga mice (25 ± 2 g) (SLC Japan, Shizuoka, Japan) were used in this experiment. They were housed in an air‐conditioned animal experiment room with a room temperature and a 50 ± 10% humidity. Before AD induction, the dorsal hair of NC/Nga mice was shaved off. There was not any sign of skin damage. AD was induced by stimulation with 2,4‐dinitrochlorobenzene (DNCB, Merck). A 1% DNCB solution (0.15 mL) dissolved in an acetone-olive oil mixture (acetone:olive oil = 3:1) was applied to the shaved dorsal skin area. After this initial sensitization treatment, the mice were dorsally treated with 0.3% DNCB at 1 week intervals for 5 weeks. The NC/Nga mice were classified into four groups; untreated, control, CS, and dexamethasone (DEX) groups. The control, CS, and DEX groups were dorsally treated with 1% DNCB and thereafter were dorsally administered with 0.3% DNCB for 12 weeks. The control, CS, and DEX groups had phosphate-buffered saline (PBS), CS extract (100, 200, 500 μg /kg), and DEX (5 mg/kg), respectively, applied to the same area of dorsal skin for 7 weeks after sensitization with 0.3% DNCB. The untreated group was treated with PBS. The severity of dermatitis was assessed macroscopically in a blinded fashion according to our previous paper (Lee et al., 2012). Experimental procedures were approved by the Institutional animal care and use committee, Eulji University.

### Histological analysis

After sacrificing the mice, the dorsal skin was separated and fixed in Carnoy's solution, embedded in paraffin (Merck) and sectioned. The tissue sections were then stained with hematoxylin‑eosin solution or alcian blue (Merck). Finally, the sections were examined by using light microscopy (Leica Microsystems, Wetzlar, Germany) for histological evaluation. For immunohistochemical staining, we performed on 4 μm-thick paraffin sections with an automated tissue staining system of Ventana Medical Systems Inc. (TuPTon, AZ, USA). The sections were placed on SuperfrostPlus microscope slides (Fisher Scientific, Madison, WI, USA). An OptiView DAIHC Detection Kit (Ventana Medical Systems) was used as a 3,3′-diaminobenzidine (DAB) for detecting antibodies. Sections were deparaffinized with EZ Prep solution. CC1 standard (Tris/Borate/EDTA, pH 8.4) was used for antigen retrieval. Slides were incubated with anti-filaggrin, anti-involucrin or anti-loricrin antibody (Santa Cruz Biotechnology) after which they were incubated with OptiView HRP Multimer, HQ Universal Linker, and H_2_O_2_. After incubating with OptiView DAB and copper, they were counterstained and post-counterstained with hematoxylin-eosin and bluing reagent, respectively.

### Measurement of serum IgE level

Blood was collected from the retro‐orbital plexus of the mice before euthanasia. Serum was obtained by centrifugation and stored at -70°C until required. Total IgE levels in the serum were measured by using a sandwich ELISA kit (R&D Systems, Minneapolis, MN, USA) and following the manufacturer's instructions.

### Splenocyte preparation

Mice were euthanized, and subsequently, their spleens were removed under aseptic conditions. Splenocytes were then isolated from the spleens after which the red blood cells were hemolyzed by using a red blood cell lysis solution (Merck). Splenocytes were seeded in a 24‑well plate at a concentration of 5 × 10^6^ cells/mL in RPMI‑1640 medium with 1% penicillin‑streptomycin and 10% fetal bovine serum (Gibco‑BRL, Grand Island, NY, USA).

### Measurement of alanine aminotransferase and aspartate aminotransferase

The concentrations of alanine aminotransferase (ALT) and aspartate aminotransferase (AST) in the serum of NC/Nga mice were measured by using the Reitman-Frankel method and ALT and AST assay kits (Asan Pharm, Seoul, Korea) according to the manufacturer's instructions.

### Statistical analysis

Values are expressed as the means ± standard deviation (SD). Intergroup differences were analyzed by applying the Student's *t*-test, using the SPSS software, version 18.0 (SPSS). A *p* < 0.05 is considered to indicate statistical significance.

## Results

### CS extract suppresses the decrease of filaggrin by blocking JNK activation due to TNF-α and IFN-γ

**Figure 1:**
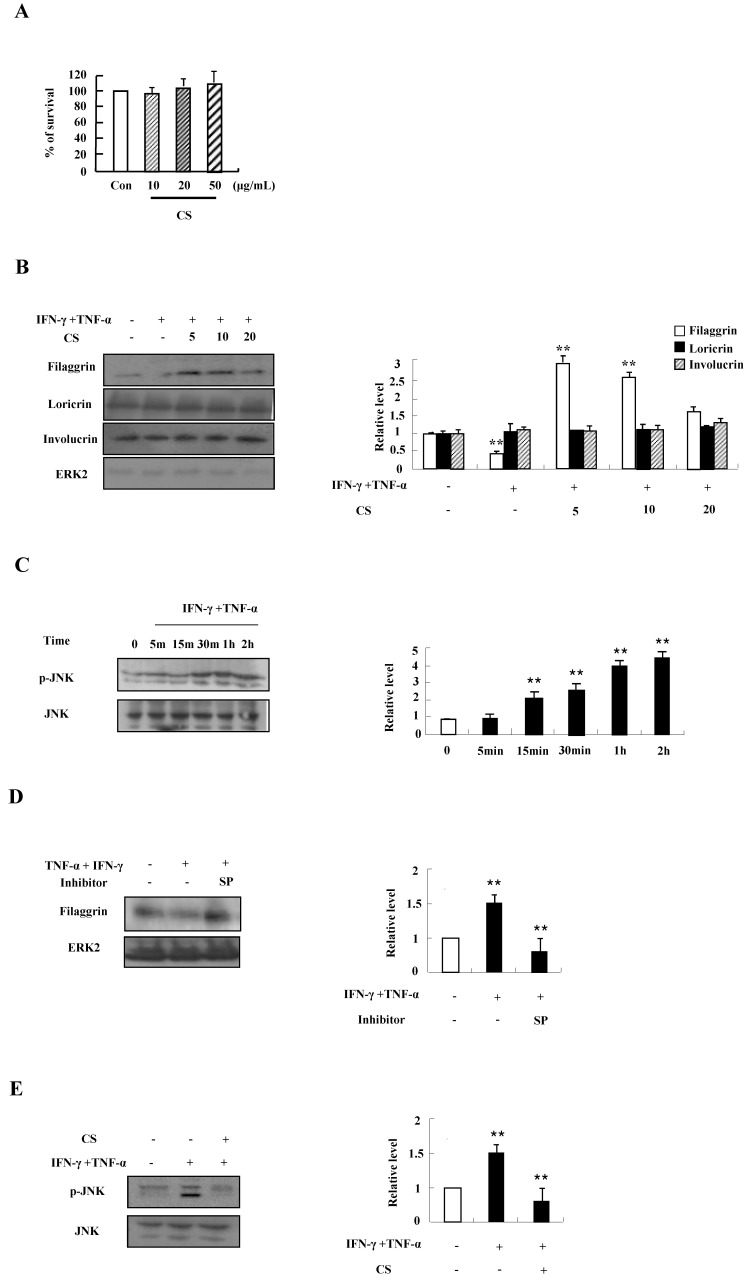
** CS recovers the decrease of filaggrin induced by TNF-α and IFN-γ treatment by inhibiting the activation of JNK. (A)** HaCaT cells were incubated in the absence (medium alone) or presence of CS extract at the indicated concentrations for 48 h. Survival rate was measured by performing MTT‑based viability assay. Data are presented as a mean ± SD of three independent experiments and expressed as a relative ratio to the absorbance of untreated cells, which was set at 100%d. **(B)** HaCaT cells were preincubated in the absence and presence of CS at the indicated concentrations for 1 h. The cells were then incubated with 10 ng/mL TNF-α and IFN-γ for 48 h. The harvested cells were lysed, and filaggrin, loricrin and involucrin were analyzed by western blotting. **(C)** HaCaT cells were treated with TNF-α and IFN-γ in a time-dependent manner. The harvested cells were lysed and phospho-JNK was analyzed by western blotting. **(D)** HaCaT cells were pretreated for 1 h with or without 20 µM SP600125 (SP), after which the cells were treated with 10 ng/mL TNF‑α and IFN-γ for 48 h. The harvested cells were lysed and filaggrin was analyzed by western blotting. **(E)** HaCaT cells were pretreated for 1 h with or without CS extract, after which the cells were treated with 10 ng/mL TNF-α and IFN-γ for 1 h. The harvested cells were lysed and phospho-JNK was analyzed by western blotting. The membrane was stripped and reprobed with anti‑ERK2 or anti-JNK antibody as an internal control. Densitometric data are expressed as a mean ± SD and are presented relative to the negative control, which was set at 1 (right panels of B, C, D and E). *P < 0.05 and **P < 0.01 indicate a significant difference between the untreated and TNF-α and IFN-γ-treated group or between the TNF-α and IFN-γ-treated group and the CS or inhibitor-treated group.

We first investigated the optimal treatment concentration of CS extract in HaCaT cells. CS extract had no cytotoxic effect on HaCaT cells because cell viability was not altered after treatment with CS extract at concentrations ranging from 10 ug/mL to 50 ug/mL for 48 h (Fig. 1A). Since alteration of skin barrier proteins is essential in the pathogenesis of AD, we investigated whether CS extract alters filaggrin expression. TNF‑α and IFN‑γ suppressed the expression of filaggrin. The decreased expression was recovered by CS extract (Fig. 1B). Expression of loricrin and involucrin was not altered by TNF‑α and IFN‑γ, and CS extract. As shown in Fig. 1C and 1D, JNK was phosphorylated by TNF‑α and IFN‑γ in a time-dependent manner, and the decreased expression of filaggrin due to TNF‑α and IFN‑γ was recovered by SP600125. Since alternation of filaggrin production by TNF-α and IFN-γ is involved in JNK activation, we examined the effect of CS on JNK phosphorylation. CS suppressed JNK activation induced by TNF-α and IFN-γ stimulation (Fig. 1E). These results indicate that CS extract may upregulate the expression of filaggrin under conditions like atopic dermatitis.

### CS inhibits the cytokine secretion of HaCaT cells

**Figure 2:**
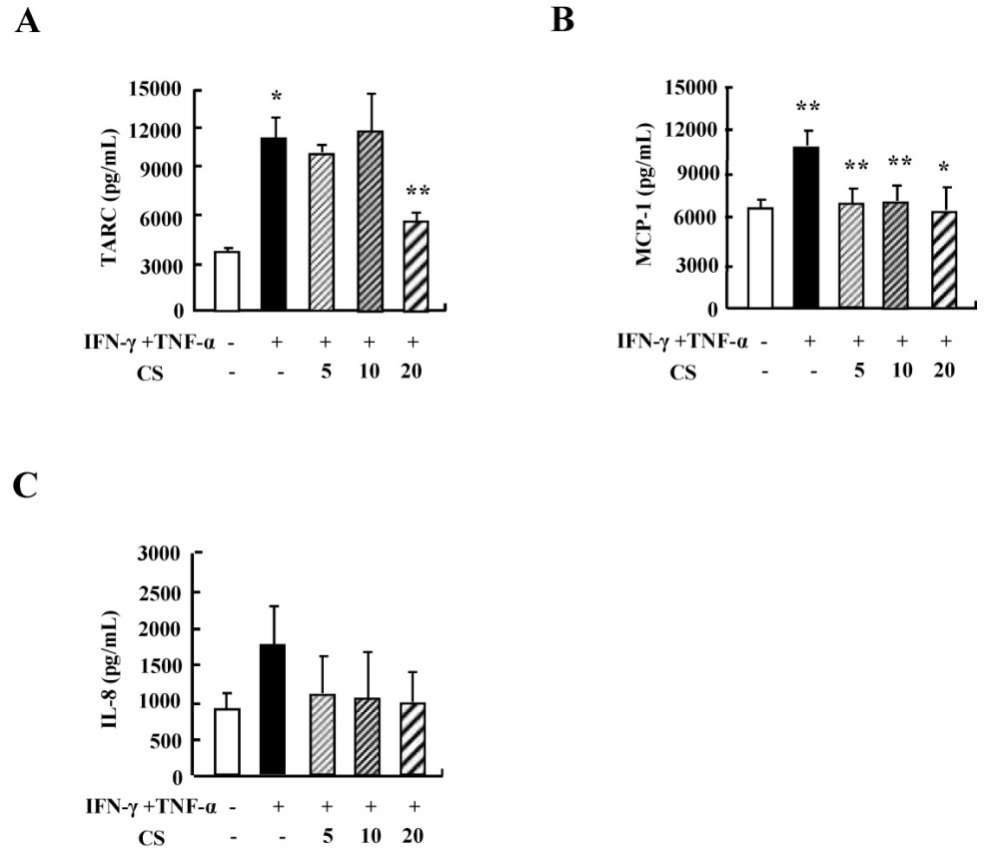
** CS suppresses the cytokine production of HaCaT cells.** HaCaT cells were pretreated in the absence or presence of CS extract at the indicated concentrations. Cells were treated with 10 ng/mL TNF-α and IFN-γ for 24 h. The supernatant was collected and analyzed by using ELISA. Data are presented as the mean ± SD of three independent experiments. *P < 0.05 and **P < 0.01 indicate a significant difference between untreated and TNF-α and IFN-γ-treated groups or between the TNF-α and IFN-γ-treated group and the CS‑treated group.

Since cytokine secretion plays an important role in inflammatory responses, we investigated the alteration of cytokine release after exposure to CS. Treatment with TNF-α and IFN-γ increased the secretion of TARC, MCP-1, and IL-8 (Fig. 2). CS decreased the release of TARC, MCP-1, and IL-8 that had been increased by IFN-γ and TNF-α stimulation.

### CS extract inhibits the aggravation of atopic-like skin lesion and decreases histopathological features in DNCB-induced AD mice

**Figure 3:**
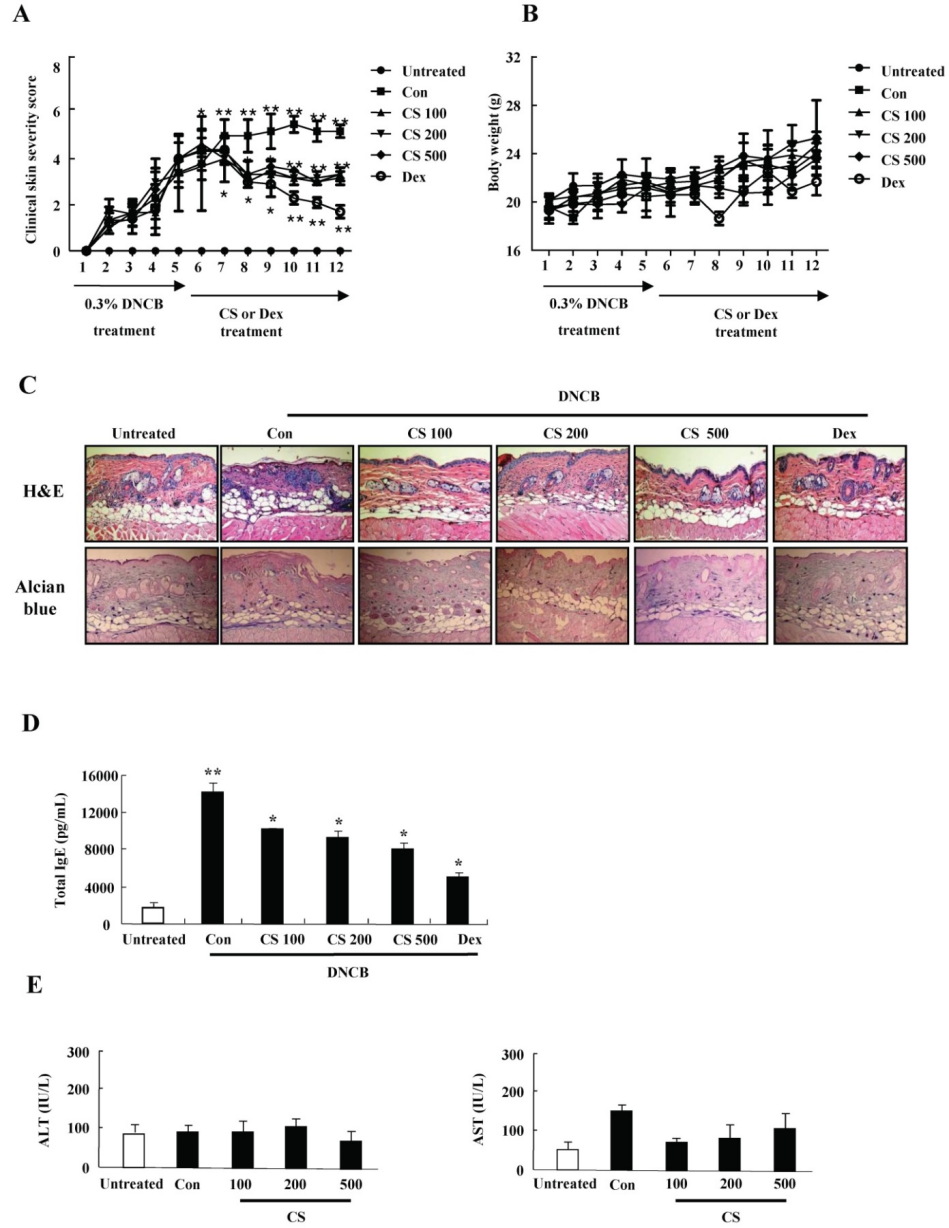
CS extract reduces the severity of atopic-like lesions and decreases the histopathological features in DNCB-induced AD mice. The mice were divided into four groups: Untreated, control (Con), CS, and DEX. The control, CS, and DEX groups were dorsally administered with 1% DNCB and then dorsally treated with 0.3% DNCB. CS was administered orally at concentrations of 100, 200, and 500 µg/kg. DEX was administered orally at 5 mg/kg. **(A)** The severity of dermatitis was evaluted macroscopically in a blinded experiment. **(B)** Mouse mean body weight was measured by using an electric scale. Data are presented as a mean ± SD. **(C)** For histological analysis, the dorsal skin was fixed, embedded in paraffin, sectioned, stained with hematoxylin-eosin and alcian blue, and examined by using light microscopy (magnification, ×100). **(D)** Total serum IgE levels were measured by using sandwich ELISA kits. **(E)** The levels of AST and ALT were measured in the serum of NC/Nga mice by using the Reitman-Frankel method and ALT and AST assay kits. Data are presented as a mean ± SD. *P < 0.05 and **P < 0.01 indicate a significant difference between the untreated and control groups or between the control and CS-treated groups.

For evaluating the suppressive effect of CS eactract in the pathogenesis of AD, we performed the pre-clinical, histological, and serological analyses. NC/Nga mice were administered with DNCB for 5 weeks and thereafter PT extract was treated to the mice for 7 weeks. CS administration recovered the increase of a skin symptom severity score due to DNCB as compared to the control group, and the score of the CS-treated group was comparable to that of the DEX-treated group (Fig. 3A). The body weight of the CS-treated group was similar to that of the control group (Fig. 3B). Histological evaluation displayed hypertrophy, hyperkeratosis of the epidermis and infiltration of inflammatory cells in the control group (Fig. 3C). However, administration of CS extract relieved the histopathological alteration in a fashion comparable to the dexamethasone group. The level of serum IgE was higher in the control group than in the untreated group, while CS treatment blocked the increased IgE concentration in serum (Fig. 3D). The serum AST and ALT were not altered in the CS-treated group as compared to those in the untreated group (Fig. 3E).

### CS extract increases the expression of filaggrin in skin of NC/Nga mice

**Figure 4:**
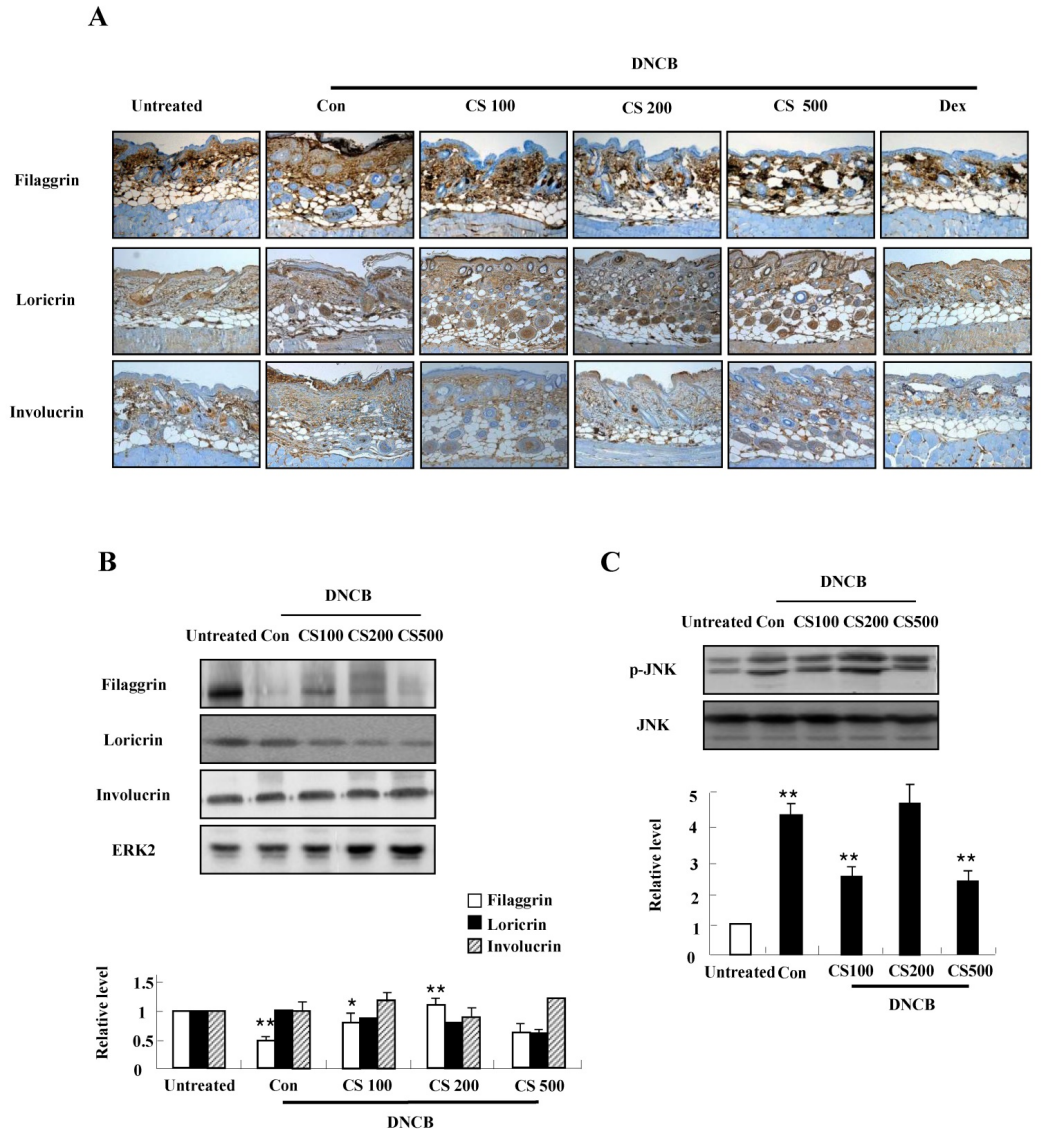
** CS extract enhances the expression of filaggrin in skin of NC/Nga mice. (A)** For filaggrin analysis, skin sections were fixed, embedded in paraffin, and stained with immunohistochemical stains. The samples were examined by using light microscopy (magnification, ×100). **(B)** Filaggrin, loricrin, and involucrin as well as phospho-JNK, in the dorsal skin were analyzed by western blotting. Densitometric data are expressed as means ± SD and are presented relative to the negative control, which was set at 1 (lower panels of B and C). *P < 0.05 and **P < 0.01 indicate a significant difference between the untreated and control groups or between the control and CS-treated groups.

We conducted both immunohistochemical staining and western blotting in order to investigate the effect of CS extract on filaggrin expression in the mice. As shown in Fig. 4A and B, CS administration recovered the decrease of filaggrin expression in epidermis. The filaggrin expression due to CS treatment was associated with inhibiting JNK phosphorylation, despite the suppression of JNK was not clearly shown in the CS (200 μg/kg)-treated group (Fig. 4C). In addition, CS extract weakly reduced the expression of loricrin and had no effect on involucrin (Fig. 4A and 4B).

### CS extract suppresses inflammatory cytokines produced from splenocytes

**Figure 5:**
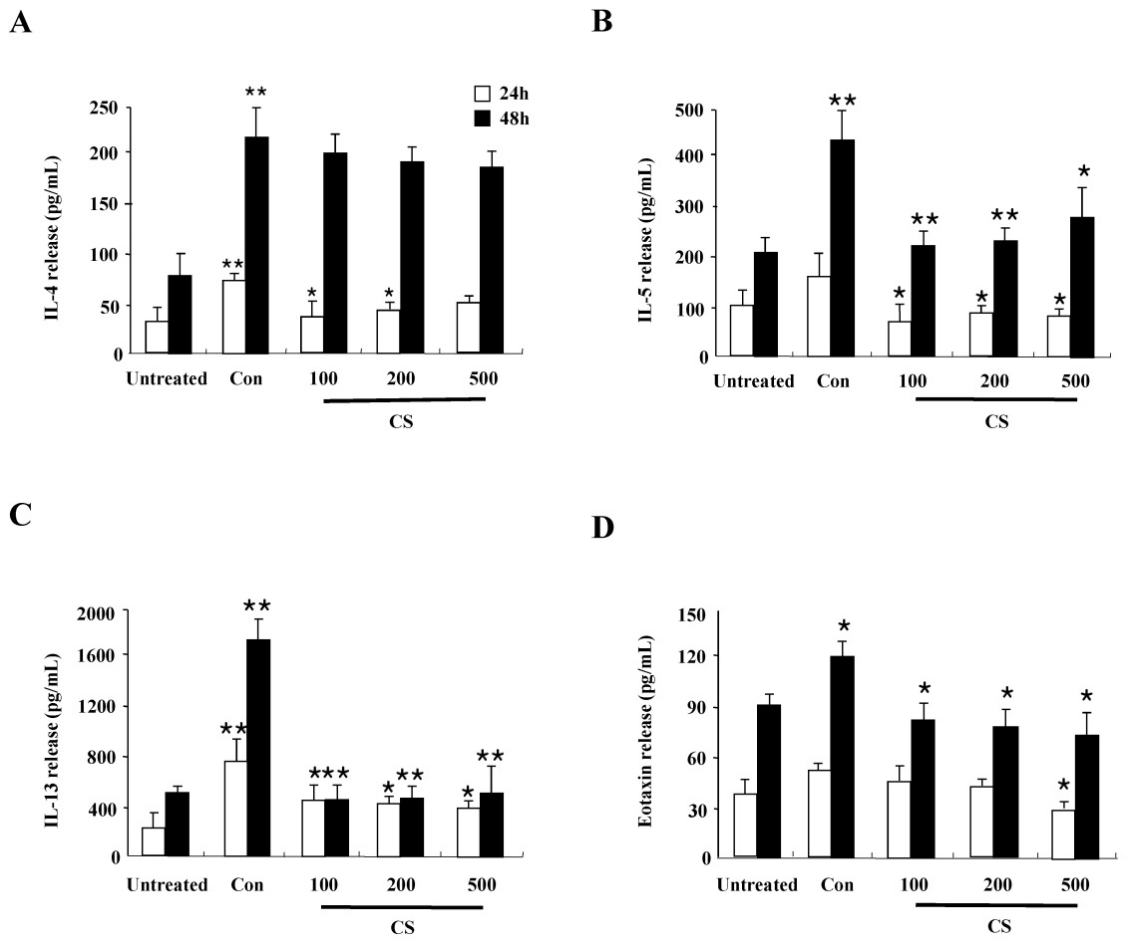
** CS extract reduces inflammatory cytokine release from splenocytes.** Splenocytes were isolated from NC/Nga mice of the untreated, control (Con), and CS groups. Subsequently, the cells were treated with 1 µg/mL concanavalin A for 24 h and 48 h. Supernatants were collected and analyzed by ELISA. Data are presented as means ± SD. *P < 0.05 and **P < 0.01 indicate a significant difference between the untreated and control groups or between the control and CS-treated groups. *P < 0.05 and **P < 0.01 indicate a significant difference between the untreated and control groups or between the control and CS-treated groups.

Since CS extract regulates cytokine production in HaCaT cells as shown in Fig. 1B, we evaluated whether the anti-inflammatory effects of CS in DNCB-induced mice is related to cytokine release of splenocytes. The synthesis of cytokines such as IL-4, IL-5, IL-13, and eotaxin increased in splenocytes of the control group, but the increased cytokines was diminished in splenocytes of the CS-treated group (Fig. 5). These results indicate that CS treatment regulates the expression of cytokines and chemokines in the clinical state of AD.

## Discussion

CS is used as a medicinal herb for the treatment of cough, pain, inflammation and itching in Korea, Japan, and China. AD, also known as eczema, is a common inflammatory skin disease and it is characterized by a variety of inflammatory responses such as cytokine production and cell infiltration into the skin 16, 17. On that basis, we investigated the anti-inflammatory effect of CS on the pathogenesis of AD and the possibility of using CS extract in a therapeutic drug for the treatment of AD.

Alteration of cytokine secretion, particularly the Th1/Th2 cytokines and chemokines, is one of the critical causes of AD 18, 19. In human keratinocytic HaCaT cells, CS extract decreased the expression of cytokines such as TARC, MCP-1, and IL-8 induced by TNF-α and IFN-γ (Fig. 2). In AD-like NC/Nga mice, the CS-treated group, after treatment with concanavalin A for 24 h and 48 h, displayed lower production of Th2 cytokines such as IL-4, IL-5, and IL-13, and a chemokine, eotaxin, than the production levels in the control group (Fig. 5). Because IL-4, IL-5, and IL-13 are involved in up-regulation of IgE production, CS may lower serum IgE level by blocking Th2 cytokine production (Fig. 3D) 20. The main chemical compositions of CS contain organic acid, triterrpenes, flavonoids and polyphenols. Oleanolic acid and ursolic acid, two major triterpene acids in CS, are effective on hepatoprotection, anti-inflammation, anti-tumor-promotion, antioxidant, and anti-hyperlipidemia 21-24. The alteration of cytokine expression in our results may be caused by these anti-inflammatory chemicals included in CS extract.

Deficiency of skin barrier proteins are essential for the development of AD. Filaggrin expression decreased by TNF-α and IFN-γ is related to activation of JNK, and CS stimulation increased filaggrin expression by blocking activation of JNK in contrast to other reports (Figs. 1 and 4) 25, 26. These results indicate that JNK is a key molecule that controls filaggrin expression. JNK is also associated with Egr-1-dependent TSLP expression induced by IL-33 and volatile organic compounds 27, 28. Because JNK is an important signal protein in the pathogenesis of AD 29, further study is required to investigate more concise mechanism related to JNK activation due to CS. Although loricrin and involucrin are two important proteins in skin barrier function, CS altered the expression of filaggrin, but was not clearly effective on loricrin and involucrin expression (Figs. 1 and 4) 30.

Nc/Nga mice were not maintained in SPF conditions for triggering AD lesion, and the mice were treated with DNCB, consistent with previous papers [31. 32]. The DNCB-treated control group showed increased clinical skin symptom severity, serum IgE level, and infiltration of inflammatory cells into skin lesions (Figs. 4 and 5). The CS-treated group displayed a low skin symptom severity score, a low serum IgE level, and alleviation of histopathological features such as movement of inflammatory cells and epidermis hypertrophy, compared to those in the control group. Body weight of CS is similar to the weight of the control group, although Dex treatment diminished the body weight of the mice. These results are comparable to the effects of other herbs and substances described in other papers [5, 33. 34]. Taken together, these results indicate that CS extract has the potential for use as a therapeutic drug for the treatment of AD.

## Abbreviations

AD: Atopic dermatitis
ALT: alanine aminotransferase
AST: aspartate aminotransferase
CS: Chaenomeles sinensis Koehne
DAB: 3,3'-diaminobenzidine
DEX: Dexamethasone
DNCB: 2,4-dinitrochlorobenzene
ELISA: Enzyme linked immunosorbent assay
IgE: Immunoglobulin E
MTT: 3-(4,5-dimethylthiazol-2-yl)-2,5-diphenyltetrazolium bromide

## Availability of data and materials

The datasets used and/or analyzed during the current study available from the corresponding author on reasonable request.

## References

[1] Nakajima S, Nomura T, Common J, Kabashima K (2019). Insights into atopic dermatitis gained from genetically defined mouse models. J Allergy Clin Immunol.

[2] Williams H, Stewart A, von Mutius E (2008). International Study of Asthma and Allergies in Childhood (ISAAC) Phase One and Three Study Groups. Is eczema really on the increase worldwide?. J Allergy Clin Immunol.

[3] Barnes KC (2010). An update on the genetics of atopic dermatitis: scratching the surface in 2009. J Allergy Clin Immunol.

[4] Kim IS, Kim DH, Yun CY (2013). A (S)-(+)-decursin derivative, (S)-(+)-3-(3,4-dihydroxy-phenyl)-acrylic acid 2,2-dimethyl-8-oxo-3,4-dihydro-2H,8H-pyrano[3,2-g]-chromen-3-yl-ester, attenuates the development of atopic dermatitis-like lesions in NC/Nga mice. Mol Biol Rep.

[5] Elhaji Y, Sasseville D, Pratt M (2019). Filaggrin gene loss-of-function mutations constitute a factor in patients with multiple contact allergies. Contact Dermatitis.

[6] Rahrig S, Dettmann JM, Brauns B (2019). Transient epidermal barrier deficiency and lowered allergic threshold in filaggrin-hornerin (FlgHrnr-/- ) double-deficient mice.

[7] Cabanillas B, Novak N (2016). Atopic dermatitis and filaggrin. Curr Opin Immunol.

[8] Cha KJ, Im MA, Gu A (2017). Inhibitory effect of Patrinia scabiosifolia Link on the development of atopic dermatitis-like lesions in human keratinocytes and NC/Nga mice. J Ethnopharmacol.

[9] Abramovits W (2005). Atopic dermatitis. J Am Acad Dermtol.

[10] Kim DH, Lee JS, Yun CY (2013). Chinese quince (Chaenomeles sinensis) extract inhibits cell migration and cytokine release in HMC-1 cells. Food and Science Biotechnology.

[11] Sawai R, Kuroda K, Shibata T, Gomyou R, Osawa K, Shimizu K (2008). Anti-influenza virus activity of *Chaenomeles sinensis*. J Ethnopharmacol.

[12] Kwon YK, Choi SJ, Kim CR (2015). Effect of Chaenomeles sinensis Extract on Choline Acetyltransferase Activity and Trimethyltin-Induced Learning and Memory Impairment in Mice. Chem Pharm Bull. (Tokyo).

[13] Zhang R, Zhan S, Li S (2018). Anti-hyperuricemic and nephroprotective effects of extracts from Chaenomeles sinensis (Thouin) Koehne in hyperuricemic mice. Food Funct.

[14] Oku H, Ueda Y, Ishiguro K (2003). Antipruritic effects of the fruits of *Chaenomeles sinensis*. Biol Pharm Bull.

[15] Han YK, Kim YS, Natarajan SB (2016). Antioxidant and Anti-Inflammatory Effects of Chaenomeles sinensis Leaf Extracts on LPS-Stimulated RAW 264.7 Cells. Molecules.

[16] Spergel JM (2010). Epidemiology of atopic dermatitis and atopic march in children. Allergy Clin North Am.

[17] Park JH, Kim MS, Jeong GS (2015). Xanthii fructus extract inhibits TNF-α/IFN-γ-induced Th2-chemokines production via blockade of NF-κB, STAT1 and p38-MAPK activation in human epidermal keratinocytes. J Ethnopharmacol.

[18] Suto H, Matsuda H, Mitsuishi K (1999). NC/Nga mice: a mouse model for atopic dermatitis. Int Arch Allergy Immunol.

[19] Nam AR, Kim DH (2016). S100A8 induces secretion of MCP-1, IL-6, and IL-8 via TLR4 in Jurkat T cells. Biomed Sci Lett.

[20] Yuk JE, Woo JS, Yun CY (2007). Effects of lactose-beta-sitosterol and beta-sitosterol on ovalbumin-induced lung inflammation in actively sensitized mice. Int Immunopharmacol.

[21] Osawa K, Miyazaki K, Imai H (1999). Inhibitory effects of Chinese quince *(Chaenomeles sinensis)* on hyaluronidase and histamine release from rat mast cells. Nat Med.

[22] Sun L, Hong Y, Guo X (1999). Studies on the chemical constituents of *Chaenomeles sinensis(Thouin.)* Koehne. Acad J Sec Mili Med.

[23] Gao L, Zhang L, Li N (2011). New triterpenoid saponins from Patrinia scabiosaefolia. Carbohydr Res.

[24] Liu S, Bai Z, Li J (2012). Comprehensive evaluation of multi-quality characteristic indexes of *Chaenomeles speciosa* and *C. sinensis* fruits. Zhongguo Zhong Yao Za Zhi.

[25] Park JH, Ahn EK, Ko HJ (2019). Korean red ginseng water extract alleviates atopic dermatitis-like inflammatory responses by negative regulation of mitogen-activated protein kinase signaling pathway in vivo. Biomed Pharmacother.

[26] Meng X, Qiu L, Song H (2018). MAPK Pathway Involved in Epidermal Terminal Differentiation of Normal Human Epidermal Keratinocytes. Open Med (Wars).

[27] Lee H, Bae HC, Kim J (2015). Chloroform upregulates early growth response-1-dependent thymic stromal lymphopoietin expression via the JNK and ERK pathways in human keratinocytes. Int J Dermatol.

[28] Ryu WI, Lee H, Kim JH (2015). IL-33 induces Egr-1-dependent TSLP expression via the MAPK pathways in human keratinocytes. Exp Dermatol.

[29] Chag KJ, Kashif A, Hong MH (2019). *Poncirus Trifoliata (L.) Raf*. Extract Inhibits the Development of Atopic Dermatitis-like Lesions in Human Keratinocytes and NC/Nga mice. Int J Med Sci.

[30] Kim BE, Leung DY, Boguniewicz M (2008). Loricrin and involucrin expression is down-regulated by Th2 cytokines through STAT-6. Clin Immunol.

[31] Im LR, Ahn JY, Kim JH (2011). Inhibitory effect of kyungohkgo in the development of 2,4-dinitrochlorobenzene-induced atopic dermatitis in Nc/Nga mice. Arch Pharm Res.

[32] Romagnani S (1994). Lymphokine production by human T cells in disease states. Annu Rev Immunol.

[33] Yang G, Lee K, Lee MH (2011). Inhibitory effects of Chelidonium majus extract on atopic dermatitis-like skin lesions in NC/Nga mice. J Ethnopharmacol.

[34] Han NR, Kang SW, Moon PD (2014). Genuine traditional Korean medicine, Naju Jjok (Chung-Dae, Polygonum tinctorium) improves 2,4-dinitrofluorobenzene-induced atopic dermatitis-like lesional skin. Phytomedicine.

